# Investigation on porous media coating plus effect of carbon quantum dots and graphite nanoparticles on tubular solar still (TSS) productivity

**DOI:** 10.1038/s41598-026-43530-8

**Published:** 2026-04-01

**Authors:** Magda K. El-Fakharany, Amr Elbrashy, Maher Rashad, Khaled Faisal Qasim

**Affiliations:** 1 Mechanical Engineering Department, Faculty of Engineering, Kafrelsheikh University, Kafrelsheikh, Egypt; 2 Mechatronics Department, Faculty of Engineering, Horus University‐Egypt (HUE), New Damietta, Egypt; 3 Production Engineering and Mechanical Design Department, Faculty of Engineering, Tanta University, Tanta, Egypt; 4 Chemistry Department, Faculty of Science, Suez University, Suez, 43518 Egypt

**Keywords:** Tubular solar still, Absorber plate coating, Nanoparticles, Thermal energy, Porous materials, Chemistry, Energy science and technology, Engineering, Environmental sciences, Materials science, Nanoscience and technology

## Abstract

Optimizing solar still design and developing cost-effective and evaporating bio-materials are critical to improving freshwater production. This study experimentally investigates the performance enhancement of a tubular solar still (TSS) through three innovative scenarios incorporating biomaterials. The first scenario involves coating the absorber plate with carbon quantum dots (CQDs) and graphite nanoparticles (GNPs). The second scenario incorporates natural porous materials: sponge and loofah on the absorber plate. The third scenario combines CQDs and sponges and compares them with conventional black paint. Performance evaluations were conducted based on hourly and cumulative water productivity and thermal efficiency. The experimental results reveal that the CQDs and sponge combination achieved the highest cumulative water productivity of 3.66 L/m^2^ and the highest thermal efficiency of 57.28%, outperforming black paint by 28.8% and conventional sponges by 3.7%. This improvement is attributed to the synergistic effect of the high heat absorption of CQDs and the porous sponge structure, which improves energy distribution and water evaporation. These results provide valuable insights for developing solar-powered desalination technologies to sustainably and efficiently produce freshwater.

## Introduction

Water is the basis of life and one of the most important resources on which human civilizations depend^1,2^. Still, according to recent statistics, more than 3.6 billion people worldwide suffer from water shortages for at least one month a year^3,4^. With the increasing population and expanding industrial and agricultural activities, providing fresh water has become one of humanity’s most significant challenges in the modern era. In contrast, solar energy is a renewable and clean source that can be exploited to address this crisis through desalination technologies, especially in areas with high solar radiation, such as the Middle East and North Africa^5,6^. Solar distillation is one of the sustainable solutions for producing fresh water at relatively low costs. This technology relies on heating water using sunlight and then condensing the steam to produce potable water^7^. Research indicates that conventional solar distillation systems produce approximately 2.05 L/m^2^/day of fresh water, while modified designs using technologies such as condensing mirrors or vacuum systems can raise productivity to 9.8 L/m^2^/day, improving up to 200%^8^. Solar stills come in various shapes to meet different needs and address challenges associated with operating conditions and manufacturing costs^9^. These shapes include simple designs such as flat and pyramidal devices, which are low-cost and easy to manufacture, making them ideal for use in rural areas with limited resources^10,11^.

On the other hand, more sophisticated shapes, such as spherical and cylindrical devices, provide higher thermal efficiency and productivity thanks to their ability to exploit solar radiation from multiple angles, making them suitable for industrial applications or areas with strong and continuous solar radiation^12–14^. In addition, specialized designs such as stepped devices increase the evaporation area, which is a good option for areas requiring greater productivity with limited resources^15,16^. The optimal choice depends largely on factors such as solar radiation intensity, available space, application objective (rural or industrial), and available budget. The best results can be achieved when the design is matched to the surrounding conditions. Al-Omari, et al.^17^ systematically review recent PV panel optimization innovations, specifically focusing on industrial and automated technologies such as solar tracking systems, robotic cleaning units, and condition monitoring tools. The literature suggests that solar distillation, including tubular solar stills (TSS), is a sustainable and environmentally friendly technology for desalination^18,19^, especially in remote areas with limited infrastructure where sunlight is abundant. However, these systems suffer from two major problems: low freshwater yield and limited thermal efficiency^20,21^. Researchers have proposed various improvements to overcome these challenges, including using nanomaterials and advanced thermal coatings and integrating systems with components such as condenser lenses, thermal storage devices, and vacuum techniques to improve vapor flow^22–25^. Sharshir, et al.^26^ focused on improving the performance and productivity of TSS systems by using nanocarbon black (CB) coated mushrooms. A series of experiments was conducted on the still system coated with different amounts of carbon black (25, 50, and 75 g/m^2^). The results showed a significant performance improvement compared to the conventional system, with the nanocoatings achieving a 62.98% improvement in water productivity for the mushroom coated with 75 g/m^2^ CB, respectively. In addition, the energy and exergy efficiency improvements reached 59.05% and 129.95% using the mushroom coated with 50 g/m^2^ CB. Five different attempts were made by Abdelaziz, et al.^27^ to improve the performance of TSS systems, focusing on engineering modifications and the use of advanced materials. They noted that v-corrugated aluminum baths have improved thermal efficiency. Also, adding a wicking material to the corrugated aluminum absorber plate enhances heat transfer and evaporation. Moreover, they reported that adding CB nanoparticles and paraffin wax to the wick under the corrugated absorber plate improves performance. The results showed that freshwater productivity, thermal efficiency, and exothermic efficiency increased by 88.84%, 221.8%, and 82.16%, respectively. Sharshir, et al.^28^ investigated the performance improvement of the TSS by combining it with thin film evaporation technology. A layer of engraved and carbonized compressed wood (MDF type) reinforced with CB nanoparticles was used. The effect of the thickness of the wood layer before carbonization on the performance was tested using three standard thicknesses (4, 6, and 9 mm). The results showed that the lowest thickness (4 mm) achieved the best productivity improvement of 30.8. Also, the CB was distributed on the engraved carbonized wood layer at different concentrations to determine the most efficient concentration. The results showed that 80 g/m^2^ concentration was the most effective, increasing the daily fresh water quantity by 67.1%. Sambare, et al.^29^ studied the integration of TSS with low-cost thermal storage materials. The experimental arrangements included three different TSS systems: jute cloth, steel pieces, and wire mesh. They showed that wire mesh achieved the highest productivity compared to all other materials. It showed an improvement in productivity of 41.35% compared to conventional solar still systems, 10.33% compared to steel pieces, and 29.78% compared to jute cloth. In the same context, Essa et al.^30^ tested filament materials such as jute and cotton, with and without nanocomposites of titanium dioxide (TiO_2_) and graphene. When using jute, the daily distilled water productivity of the modified system improved by 92.5% compared to the conventional system, with conventional productivity reaching about 7900 ml/m^2^/day compared to 4100 ml/m^2^/day for TSS. When jute was combined with nanocomposites, the productivity increased by 114% to reach 9000 ml/m^2^/day compared to 4200 ml/m^2^/day for the conventional system. For the use of cotton filament, the productivity of the modified system increased by 88% and 107% when used without and with nanocomposites, respectively. On the other hand, the tubular structure with an internal heat exchanger proved effective in accelerating the distillation process. Adopted as a phase change material (PCM) showed its effectiveness as a latent heat storage medium, which improved the freshwater yield and overall system efficiency^31,32^. Amin et al.^33^ modified a TSS model that integrates a heat exchanger and PCM-based system using a parabolic solar concentrator. The simultaneous integration of the heat exchanger and the PCM resulted in significant improvements, with the water yield reaching 8.06 L/m^2^/day and the system’s thermal efficiency reaching 54.04%. Environmental, economic, and exergonic analysis are essential tools for evaluating the effectiveness of solar distillation systems and analyzing their long-term sustainability. Several studies have covered these aspects, which have confirmed that using solar energy in the distillation process is a sustainable and environmentally friendly option^34^. Also, by incorporating these improvements, energy consumption costs can be reduced, and economic returns can be increased by increasing the amount of water produced without the need for large investments in infrastructure. Exergonic efficiency is improved through techniques such as improving the design of heat-absorbing surfaces and using PCM, which enhance the system’s efficiency in using renewable energy and reducing exergonic losses^35–37^. Recent studies show that nanomaterials such as carbon quantum dots (CQDs) and nano-graphite (GNPs) can improve solar energy absorption, which increases thermal conversion efficiency within distillation systems^38^. The thermal photovoltaic panel is one of the strategies that enhance energy conversion, thermal regulation, and freshwater output^39,40^. The efficiency of photothermal conversion strongly governs the performance of solar stills, the ability of the absorber surface to capture solar radiation and convert it into usable thermal energy^41^. When these doped materials are combined with porous structures such as natural sponges or loofah fibers, the synergistic effect enhances light harvesting, improves energy distribution across the surface, and accelerates evaporation rates. These characteristics justify the selection of nanomaterial–biomaterial combinations as a promising strategy for TSS performance improvement^42^.

Based on previous studies, conventional TSS are simple and energy-efficient solutions that rely on solar energy to produce pure water. However, these systems face significant thermal efficiency and productivity limitations, which limit their economic feasibility on a large scale. Ongoing efforts to improve the efficiency of TSS using technologies include engineering designs, adding fins, reflectors, nanomaterials, and PCM^43–45^. Yu et al.^46^ demonstrated the microplastic removal efficiency was enhanced by up to 5.5 times compared with previously reported adsorbent-based approaches, establishing a new benchmark for water-quality-oriented solar desalination systems. Yu et al.^47^ reported a lithium extraction capacity of ~ 34 mg g⁻^1^ (HMO basis), exceeding that of conventional lithium-ion sieve powders, while solar irradiation enhanced the extraction kinetics by 2.9-fold compared to dark conditions. Liu et al.^48^ achieved a high evaporation rate of 3.51 kg m⁻^2^ h⁻^1^ under 1 sun and stable operation in 20 wt% saline brine for over 24 h without salt accumulation. The study demonstrated an evaporation rate exceeding ~ 1.5–2.0 kg m⁻^2^ h⁻^1^ under 1 sun, with enhanced stability attributed to optimized pore networks that minimized heat loss and improved capillary water transport. Zhang, et al.^49^ further explored the coupling of photothermal materials with engineered porous substrates to improve vapor transport and salt rejection.^50^ demonstrated that combining photothermal absorbers with tailored three-dimensional architectures can significantly improve both evaporation rate and operational stability. It is provided experimental evidence that evaporation enhancement arises from the combined effects of solar absorption, capillary-driven water supply, and reduced conductive heat losses.

While previous studies have explored nanomaterial coatings or porous/wicking structures independently, and a limited number have combined them, our work is distinct in three key aspects:

- The use of carbon quantum dots (CQDs) as the primary photothermal agent in a tubular solar still configuration, which has not been systematically investigated previously.
- The explicit comparison of three absorber modification strategies (nanomaterial-only, porous-only, and hybrid nanomaterial–porous), under identical geometrical and climatic conditions, enabling isolation of synergistic effects.
- The demonstration that hybrid CQDs–sponge absorbers outperform both nanomaterial-only and porous-only systems, with quantified gains in productivity and thermal efficiency.

## Methods and materials

This study experimentally investigates the performance enhancement of TSS through three innovative scenarios incorporated with bio-materials. The first scenario involves coating the absorber plate with CQDs and GNPs. The second scenario incorporates bio-porous materials like a sponge and a loofah on the absorber plate. The third scenario combines CQDs and sponges, all of which are compared with conventional black paint.

### Preparation and characterization of nanomaterials

**Fig. 1:**
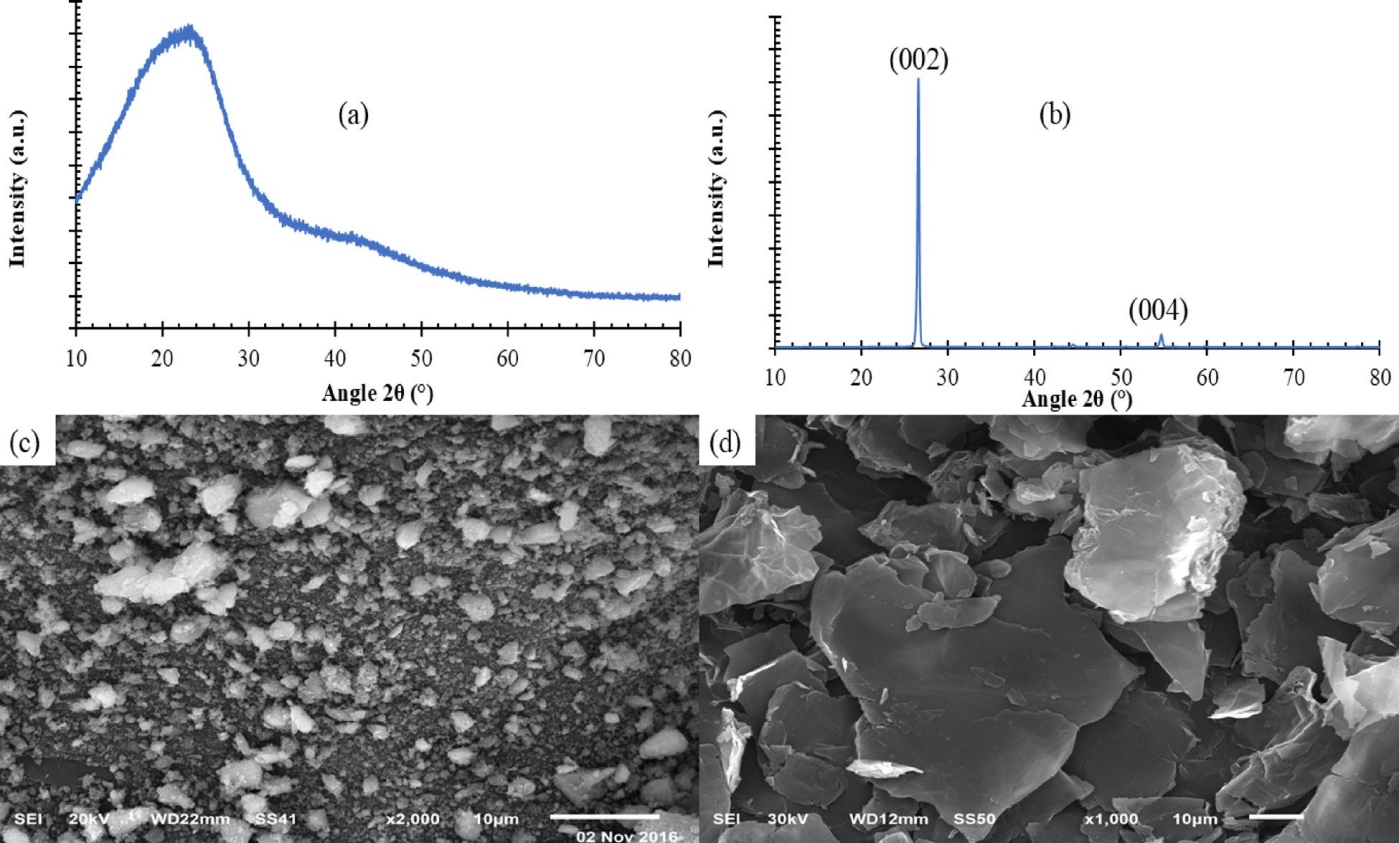
XRD of (**a**) carbon quantum dots and (**b**) nano graphite, and SEM image of (**c**) carbon quantum dots and (**d**) nano graphite.

CQD and GNPs represent an advanced generation of nanomaterials with unique properties that make them ideal for enhancing solar distillation performance. CQDs are nanosized carbon particles that exhibit exceptional optical and thermal properties. Their most notable features include their ability to absorb a wide spectrum of solar radiation and efficiently convert it into heat, their high stability in aqueous solutions, and their environmental friendliness. GNPs have very high thermal conductivity, which enhances heat transfer to the water within the distillation system. Thanks to their relatively large surface area at the molecular level, GNPs improve solar energy absorption and heat distribution more efficiently, accelerating evaporation and increasing freshwater productivity. CQD was prepared by pyrolysis. Without a solvent, citric acid was added directly to an oven and pyrolyzed for 15 h at 210 °C. The resultant powder was washed with deionized water and centrifuged to obtain the consequent powder . While GNPs were prepared from 95% graphite (mesh size 1–10 μm), citric acid (C6H8O7) and sodium hydroxide (NaOH) were purchased from Nice, India. The morphological behavior of nanoparticles (CQDs, GNPs) is studied by subjecting them to SEM and XRD. XRD patterns are presented in Fig. 1a,b. As shown in the figure, the XRD analysis revealed that two diffraction peaks are occurring at 26.3° 44.7° for CQDs and 26.3° and 44.7° related to (002), and (004) planes for GNPs. Figure 1c,d shows the SEM image of the synthesized CQDs and GNP nanoparticles. The image shows clearly that the CQDs nanoparticles have spherical morphology with a grain size of 5 nm. In comparison, GNP nanoparticles have a plate morphology with a grain size of 50 nm. The XRD patterns for both CQDs and GNPs illustrate the presence of distinct crystalline phases associated with graphitic carbon structures, reinforcing their capability to absorb and dissipate radiant energy effectively. The CQD pattern, dominated by broad peaks, indicates a partially amorphous structure rich in defect sites, which is advantageous for multiple scattering and enhanced photon absorption. The GNP peaks, sharper and more crystalline, reflect improved thermal pathways across the material. These interpretations are supported by the SEM micrographs, which show nanoscale spherical CQDs and plate-like GNPs. The small size of CQDs allows uniform surface distribution, increasing the effective surface area exposed to radiation, while the layered morphology of GNPs facilitates lateral heat spreading. These structural features directly inform the thermal behavior observed in the experimental results.

The properties of carbon CQDs and GNPs are displayed in Table 1. This table captures the key thermophysical properties of CQDs and GNPs, highlighting their distinct roles in thermal management and solar energy absorption applications^51^.

**Table 1:** Thermophysical Properties of the tested nanoparticles.

Property	Carbon quantum dots (CQDs)	Graphite nanoparticles (GNPs)
Thermal conductivity (W/m·K)	N/A	50–100
Specific heat capacity (J/g·K)	0.3–1.8	0.71
Density (g/cm^3^)	0.8–2.2	2.2
Thermal stability (°C)	80	600
Size (nm)	5	50–200

### Experimental setup and procedure

**Fig. 2:**
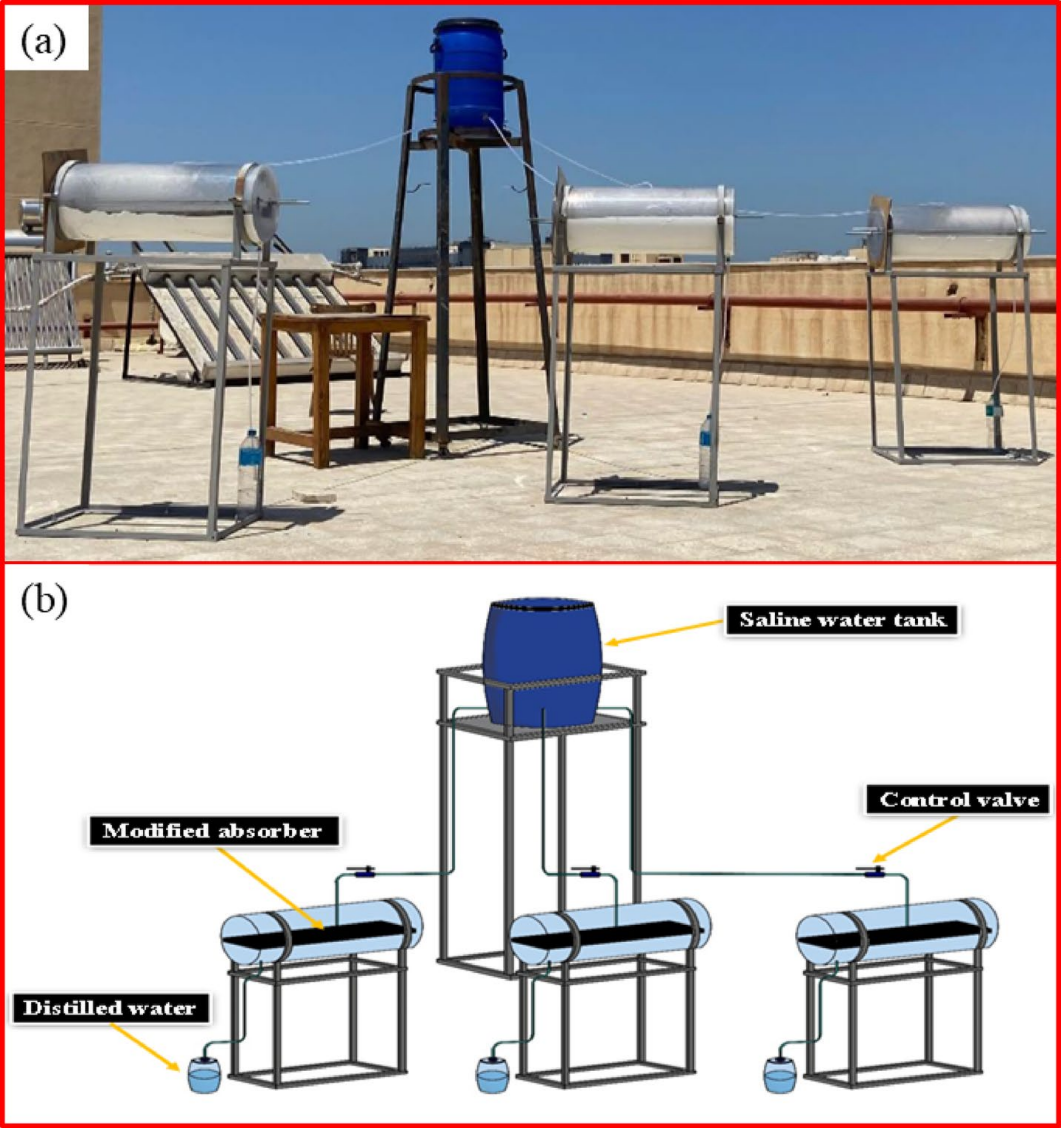
(**a**) Experimental photograph, (**b**) Layout diagram of experimental setup.

The introduced TSS design consisted of a transparent polycarbonate cylinder for effective thermal transparency. A transparent tube, a container containing the salty water to be treated, a heat-absorbing surface, and a condensation path. Also, the internal basin’s surface was coated with black paint to optimize its absorptivity and prevent the reflection of solar radiation.. Experiments were conducted using three TSSs with the same design, materials, and construction, but with different absorber plate coatings. The TSSs are fabricated from polycarbonate sheets, which are 2 mm thick. Each TSS has a diameter of 30 cm and a length of 75 cm. The absorber plate has a rectangular shape with dimensions of 70 cm, 25 cm, and 0.15 cm (length, width, and thick), and 1 cm side wall height. Each basin contained 375 mL of saline water, which was kept constant during the experiments. It was fed through attached feeding pipes to keep the basin water constant. Everything for the three TSSs is the same except the coating of the absorber plate to ensure fair comparisons. A photograph of an experimental setup is shown in Fig. 2a, and a layout is illustrated in Fig. 2b. Proper representation of the water flow path is essential for interpreting heat and mass transfer behavior inside the tubular still. The updated schematic clarifies the feed-water entry, heating zone distribution, and condensate collection route. This aids in understanding how uniform water depth, controlled inflow, and optimized surface contact enhance evaporation stability and reduce temperature fluctuations. The flow path also influences vapor movement direction, condensation onset, and thermal boundary layer development along the transparent cover. Such details justify the improvements observed when using porous materials or nanocoated absorbers.

To ensure the reliability and repeatability of the experimental results, each coating scenario was tested under identical operating conditions over multiple experimental runs. Specifically, three independent experimental repetitions were conducted for each configuration (conventional black paint, nanomaterial-based coatings, porous media coatings, and hybrid coatings). Each experimental run was carried out over a full daylight operating period (from 9:00 to 17:00 local time), covering the complete solar irradiation cycle. The reported hourly and cumulative freshwater productivity, absorber temperature, and thermal efficiency values represent the average of the repeated experiments, while the observed variations remained within the calculated experimental uncertainty limits. This repetition strategy minimizes the influence of transient meteorological fluctuations and experimental noise, thereby enhancing the statistical reliability and robustness of the comparative performance evaluation.

**Fig. 3:**
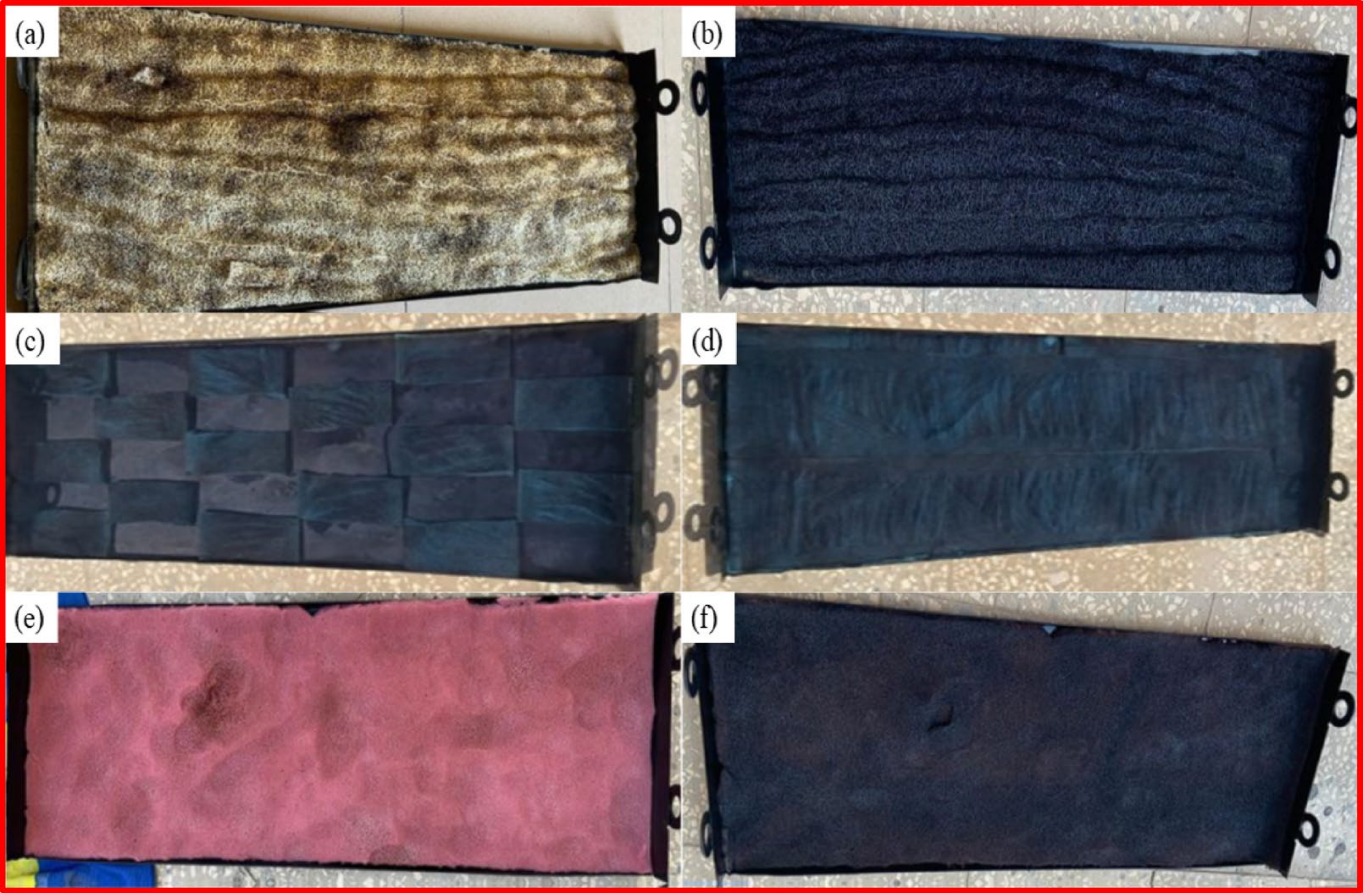
Preparation of coating materials. (**a**) carbonized loofah, (**b**) loofah with black paint, (**c**) sponge pieces with carbon quantum dots, (**d**) sponge with carbon quantum dots, (**e**) sponge and (**f**) sponge with black paint.

The experiments were conducted in Kafrelsheikh city, Egypt (31º 05′ 54′′ N and 30º 57′ 00′′ E). The idea of TSS work is that when sunlight shines on a transparent tube, the rays pass through and are absorbed by the black surface or heat-absorbing base. The salty water is then heated inside the system, causing it to evaporate. Contaminated water evaporates, leaving salts and solid impurities behind. The steam rises inside the tube and reaches a cooler interior surface due to the temperature difference between the inside and outside. The steam condenses to form pure water droplets, then slides down the inclined internal surfaces towards the collecting duct. The remaining salts and impurities are emptied from the raw water container periodically. Experiments were conducted using nanomaterials CQDs and GNPs, sponges, and loofah as coating materials on an absorber plate. The produced nanoparticles are initially selected at concentrations of 5% by weight and mixed in the black paint. Then, the CQDs and GNPs in black paint are added and mixed homogeneously in a magnetic stirrer for 0.5 h. Further to the stirring operation, the black paint with NP’s is ultrasonicated for one hour. A spray gun technique is used to coat the absorber plate. In terms of absorber coating, three scenarios were investigated. Scenario 1: coating absorber plate with nanomaterials (CQDs, GNPs), scenario 2: coating absorber plate with sponge and loofah, scenario 3: coating absorber plate with sponge and CQDs. Figure 3a–f presents the different coating absorbers for the experimental scenarios.

### Measurement and uncertainty

The ambient temperatures, cover, saltwater, and desalinated water are all measured during each investigation in the current experimental work. The temperatures are measured using a digital thermometer, type K. The intensity of solar radiation is measured using a digital solar power meter with a range 1–3999 W/m^2^. The wind speed is measured using a digital anemometer with a 0 to 30 m/s scale. The volume of desalinated water is recorded every hour using a 500 mL graduated bottle. A 5-L graduated cylinder with an accuracy of ± 105 mL was utilized, while the hourly uncertainty in freshwater measurement was approximately ± 3 mL. 

 Table 2 presents the measurement range, accuracy, and uncertainty levels for the various devices used in this study, including experimental sensors and their precision. The total uncertainty of any function X: Where ∂x_1_, ∂x_2_ ∂x_3_ … ∂x_n_ are feasible in × 1, × 2,… xn measurements_._ ∂U is absolute uncertainty, where ∂ xi denotes possible measurement errors^52,53^.

1 $$U={\sqrt{{\left(\frac{\partial U}{\partial {{\boldsymbol{x}}}_{1}}{\boldsymbol{\delta}}{{\boldsymbol{x}}}_{1}\right)}^{2}+{\left(\frac{\partial U}{\partial {{\boldsymbol{x}}}_{2}}{\boldsymbol{\delta}}{{\boldsymbol{x}}}_{2}\right)}^{2}+\dots +{\left(\frac{\partial U}{\partial {{\boldsymbol{x}}}_{{\boldsymbol{n}}}}{\boldsymbol{\delta}}{{\boldsymbol{x}}}_{{\boldsymbol{n}}}\right)}^{2}}}$$

where ∂ × 1, ∂ × 2 ∂ × 3 …: ∂xn are feasible uncertainty in × 1, × 2,… xn measurements. ∂U is known as absolute uncertainty, where ∂ xi denotes possible measurement errors.

**Table 2:** The uncertainty of measurement.

Device	Uncertainty	Accuracy	Range
Thermocouples	1	± 1 °C	− 55 to 180 °C
Solar radiation meter	0.45	± 10 W/m^2^	1–3999 W/m^2^
Digital anemometer	2.7	± 0.5 m/s	0.1–30 m/s
Graduated bottle	2	–	500 ml

### Thermal efficiency

Thermal efficiency is a key performance indicator that quantifies the ability of a solar still to convert incident solar radiation into useful thermal energy for water evaporation. This study evaluated thermal efficiency across three innovative configurations of a TSS, each designed to enhance solar energy absorption and evaporation performance. Thermal efficiency was evaluated both on an hourly and daily basis using the following equations^31^:

- *Hourly Efficiency:*

2 $${\eta }_{th,h}=\frac{{m}_{h.}LH}{3600\times {I}_{avg,h. }\left({A}_{TSS}\right)}$$

- *Daily Efficiency:*

3 $${\eta }_{th,day}=\frac{{m}_{day. }LH}{\Delta t{I}_{avg, day. }.\left({A}_{TSS}\right)}$$

where ${m}_{h}$.​ and ${m}_{day.}$.​ are the hourly and daily collected water masses, respectively, and $LH$ is the latent heat of evaporation collected during the day, ${{\boldsymbol{m}}}_{{\boldsymbol{h}}}$ is the total mass collected during 1 h , and ${\boldsymbol{L}}{\boldsymbol{H}}$ is the latent heat as a function of water temperature, Eq. (4).

4 $$LH=\left\{\begin{array}{c}3261500\left(1-7.616\times {10}^{-4}{T}_{w}\right)\to {T}_{w}>70\\ 2493500\left(1-9.475\times {10}^{-4}{T}_{w}+1.313\times {10}^{-7}{T}_{w}^{2}-4.795\times {10}^{-9}{T}_{w}^{3}\right)\to {T}_{w}<70\end{array}\right.$$

### Exergy analysis

To complement the thermal efficiency evaluation, an exergy analysis was carried out to quantify the quality of energy conversion within the tubular solar still (TSS) under the examined scenarios. Exergy represents the maximum useful work obtainable when a system is brought into equilibrium with its environment. For solar distillation, exergy analysis provides deeper insight into the irreversibilities associated with heat transfer, evaporation, and condensation processes.

The exergy input from solar radiation was calculated using the Petela model:

5 $$E_{{{\mathrm{solar}}}} = A_{{{\mathrm{TSS}}}} \,I\left[ {1 - \frac{4}{3}\frac{{T_{0} }}{{T_{s} }} + \frac{1}{3}\left( {\frac{{T_{0} }}{{T_{s} }}} \right)^{4} } \right]$$

where, ${A}_{TSS}$ is the absorber surface area, $I$ is the measured solar intensity, ${T}_{0}$ is the ambient temperature (K), and ${T}_{s}=5777$ K is the effective temperature of the sun.

The exergy associated with evaporated water was calculated from the specific exergy of phase change:

6 $$E_{{{\mathrm{evap}}}} = m_{{\mathrm{w}}} \left[ {L_{H} \left( {1\frac{{T_{0} }}{{T_{w} }}} \right)} \right]$$

where ${m}_{w}$ is the mass of distilled water, ${L}_{H}$ is the latent heat of evaporation, and ${T}_{w}$ is the water temperature (K).

The exergy efficiency of the solar still was determined as:

7 $${\eta }_{\mathrm{ex}}=\frac{{E}_{\mathrm{evap}}}{{E}_{\mathrm{solar}}}$$

Uncertainty propagation was applied to all calculated exergy terms using:

8 $${U}_{E}=\sqrt{{\left(\frac{\partial E}{\partial {T}_{0}}{U}_{{T}_{0}}\right)}^{2}+{\left(\frac{\partial E}{\partial I}{U}_{I}\right)}^{2}+{\left(\frac{\partial E}{\partial {m}_{w}}{U}_{m}\right)}^{2}+{\left(\frac{\partial E}{\partial {T}_{w}}{U}_{{T}_{w}}\right)}^{2}}$$

where ${U}_{X}$ represents the uncertainty of each experimental measurement.

This approach provides a comprehensive assessment of the system’s thermodynamic performance, enabling comparison between nanomaterial-coated, porous, and hybrid absorber configurations.

## Results and discussions

In this section, the results obtained through the experiment are presented to evaluate the performance of the TSS system. The performance of the materials used, such as sponge and loofah sprayed with CQDs and GNPs, is analyzed, emphasizing their impact on thermal efficiency, water productivity, and surface temperatures. The results are presented using three integrated scenarios, comprehensively comparing conventional and improved systems using porous surfaces and nanomaterials. The results are discussed from a physical and practical perspective, explaining the impact of design modifications and innovative materials on performance improvement.

### Coating absorber with nanomaterials

**Fig. 4:**
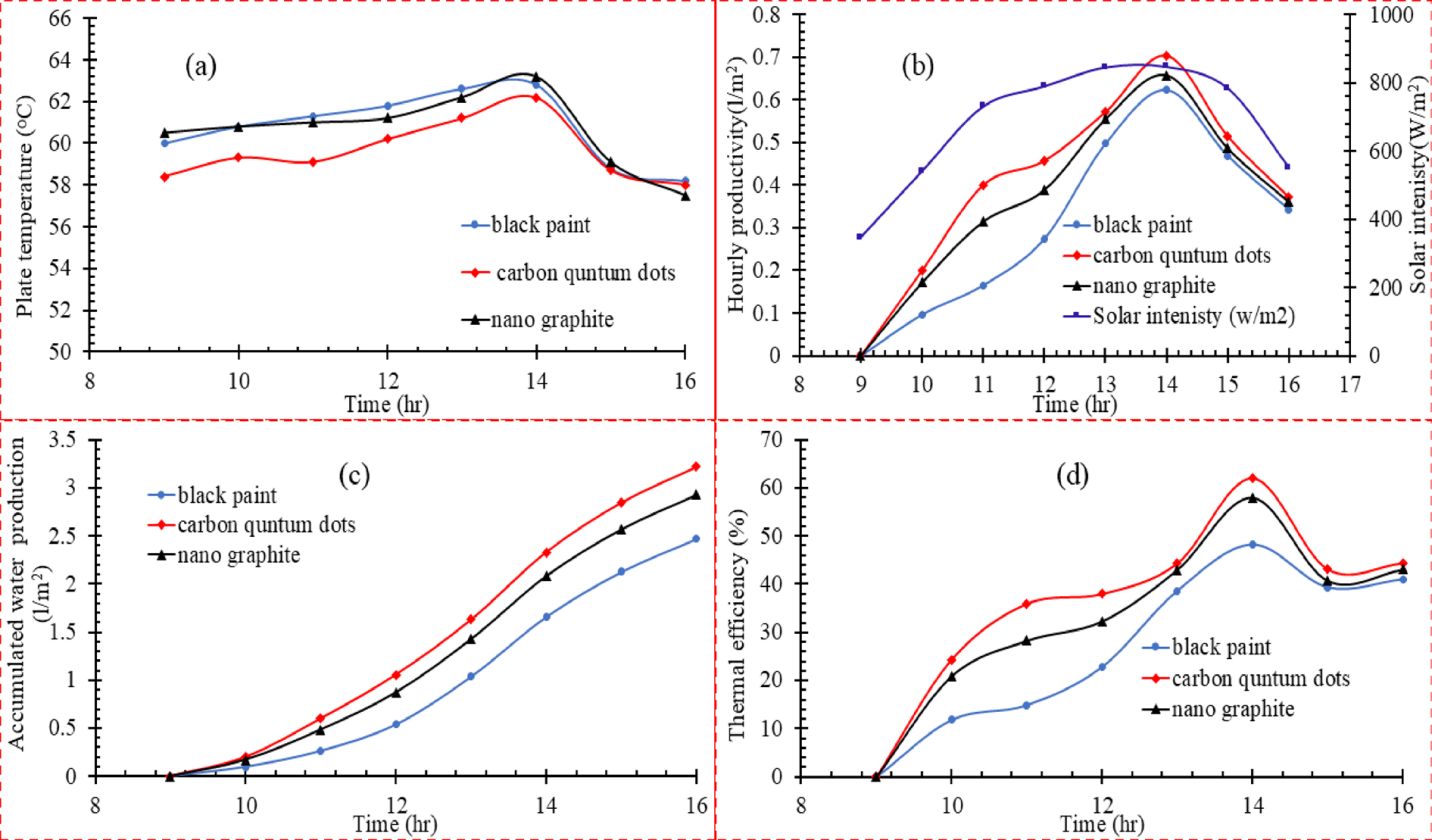
(**a**) Influence of absorber plate coating on (**a**) the plate temperature, (**b**) water production, (**c**) accumulated water production in the experimental period, (**d**) thermal efficiency in (scenario 1).

As illustrated in Fig. 4a**,** the black coating with GNPs outperformed the black coating in achieving the highest average temperature of 61.5°C, indicating its high efficiency in absorbing solar radiation and converting it to heat. The highest peak temperature of 63.8°C indicates its excellent performance during maximum solar radiation. The black coating with CQDs showed the lowest surface temperature (59°C average temperature), possibly due to the higher heat transfer to basin water due to their ability to absorb a wide spectrum of solar radiation. The black coating with GNPs showed performance very close to that of the black coating. This performance outperforms the CQDs (about 3.9% higher average temperature), reflecting its potential to achieve efficient heat absorption while potentially improving operational costs.

On the other hand, Fig. 4b shows hourly distilled water productivity against time at different absorbing coating types. The black coating showed the lowest peak yield compared to the different materials (0.55 L/m^2^/hr), indicating a lower thermal energy absorption and conversion efficiency than GNPs and CQDs. The carbon quantum dot coating performed well at the peak (0.70 L/m^2^/hr), making it a reasonable economic choice. In contrast, the GNPs coating showed a yield (0.65 L/m^2^/hr). The graph shows a gradual increase in productivity until the peak is reached, indicating the stability of the material in converting thermal energy into productivity. It is worth noting that all materials showed a peak yield at 14:00, coinciding with the highest solar radiation intensity (900 W/m^2^). The cumulative productivity of the three surfaces was observed in Fig. 4c to be 2.46 L/m^2^, 2.93 L/m^2^, and 3.21 L/m^2^ for black coating, GNPs, and CQDs, respectively. Compared to the black coating, CQDs show an increase in productivity by 30.49%, and GNPs show an improvement of 19.11%, indicating their acceptable efficiency in heat absorption and conversion into productivity. CQDs can be considered a promising material for improving the performance of solar thermal systems. The thermal efficiency of the three types of absorbers shown in Fig. 4d was analyzed at 14:00. The results showed that CQDs had a clear superiority with a thermal efficiency of 61.9%, followed by GNPs with 58%, and black coating with 48.18%. The superior performance of CQDs is attributed to their excellent properties in effectively absorbing solar radiation due to their fine nanostructure, which contributes to increasing the conversion of solar energy into useful thermal energy while reducing heat loss by radiation. GNPs performed well due to their high thermal conductivity and uniform heat distribution across the absorber surface, which reduced the loss due to uneven heat conduction. In contrast, black coating came in last place due to its limited heat storage capacity and the significant heat loss by radiation and conduction, significantly reducing its thermal efficiency compared to nanomaterials. The integration of porous biomaterials and nanomaterials modifies the heat–mass transport mechanism inside the still. Porous surfaces create micro-scale liquid films, improving the evaporation-to-surface-area ratio. Nanocoatings increase absorptivity while reducing reflectivity. Together, these mechanisms accelerate vapor generation even under moderate solar flux. Furthermore, the porous–nanomaterial composite creates hybrid photothermal surfaces that sustain evaporation over longer periods due to enhanced heat retention and capillary refilling. This hybridization explains why Scenario 3 consistently outperformed all other configurations in both thermal efficiency and cumulative productivity.

### Coating the absorber with a sponge and a loofah.

**Fig. 5:**
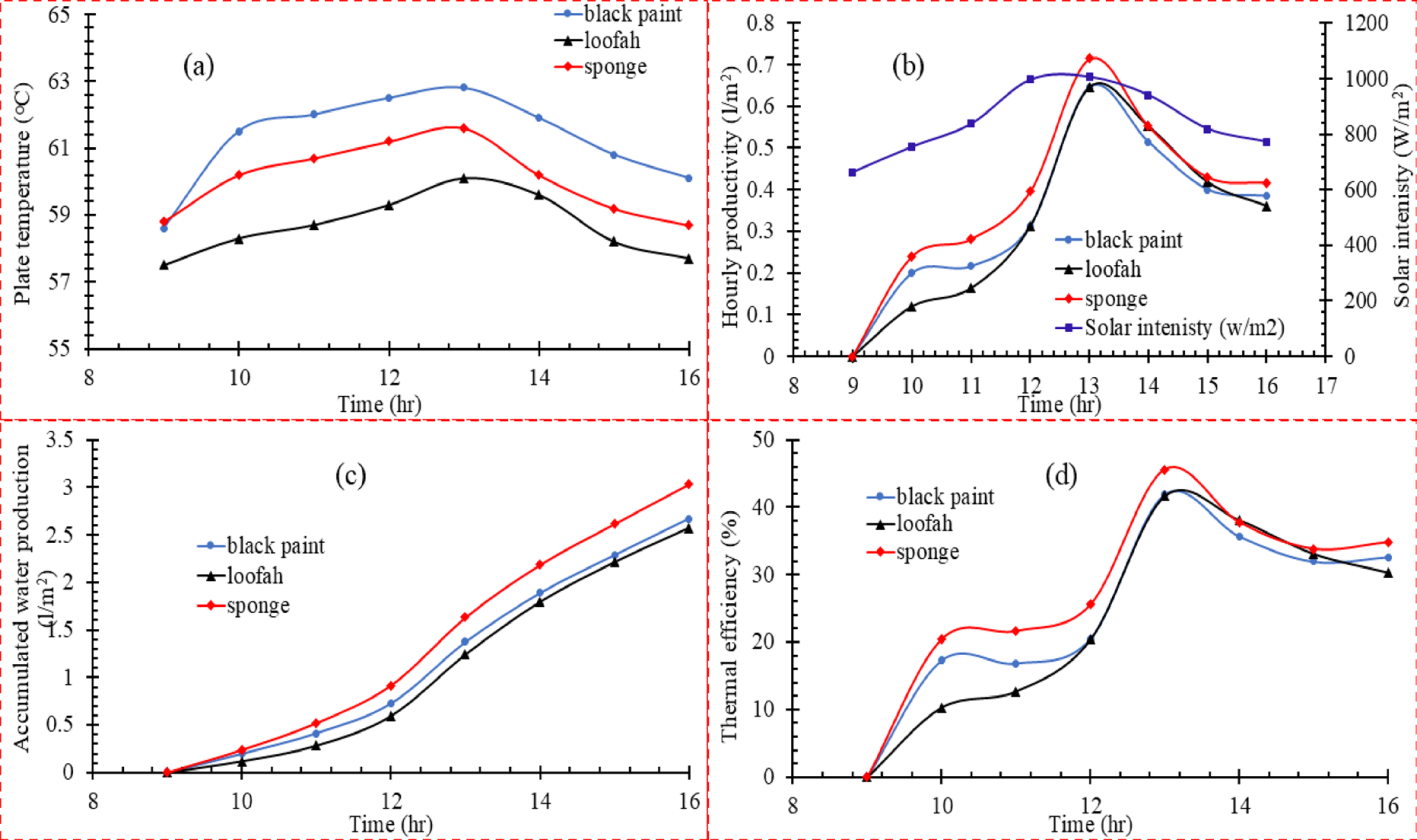
(**a**) Influence of absorber plate coating on (**a**) the plate temperature, (**b**) water production, (**c**) Accumulated water production in the experimental period, (**d**) thermal efficiency in (scenario 2).

In this second scenario, the performance of three types of materials on the absorber surface was compared: black paint, loofah, and sponge, to evaluate the effect of these materials on the temperature, productivity, thermal efficiency, and cumulative productivity during the experiment time of the solar desalination system. The surface covered with black paint recorded the highest temperature at 13:00, reaching 62.8 °C, outperforming the sponge at 61.6 °C. In comparison, the loofah recorded the lowest temperature at 60.1 °C, as shown in Fig. 5** a**. These results can be explained by the fact that black paint has a higher capacity to absorb solar radiation and convert it into heat than other materials, which contributes to reaching the highest temperature on the absorber surface. However, although the sponge does not achieve the highest temperature, it remains effective in water desalination applications due to its porous properties that enhance heat transfer in the system. Despite its efficiency in absorbing heat, the loofah does not show the same superior performance compared to the black paint and sponge, which translates into lower temperatures. As for hourly water productivity, as shown in Fig. 5b the sponge recorded the best performance with a productivity of 0.71 l/m^2^/h, outperforming the loofah 0.64 l/m^2^/h and the black paint 0.63 l/m^2^/h. This result can be explained by the sponge’s ability to maintain a higher temperature for a longer period, increasing the evaporation rate on its surface. While the loofah and the black paint were in the same range, reflecting a very close performance in converting heat into water vapor. This is because the used loofah is uncompressed and has high porosity and low thermal conductivity. The porous structure of the sponge increased its interaction with solar rays. It achieved the highest productivity, which made its performance outperform that of black paint and loofah. As for the cumulative productivity in Fig. 5c the sponge recorded the highest value with a cumulative productivity of 3.02 l/m^2^ throughout the experiment. In contrast, the black paint recorded 2.67 L/m^2^ and the loofah 2.57 L/m^2^, indicating that the sponge outperforms in instantaneous productivity and total productivity over the working hours. The gap between the loofah and the black paint was small in cumulative productivity, reflecting their balanced performance in this context.

Regarding thermal efficiency, Fig. 5d shows the highest thermal efficiency of the sponge at 45.57%, followed by the black paint at 41.97%, and then the loofah at 41.63%. These results reflect the sponge’s ability to convert solar energy into heat and use it more efficiently than other materials. Although the black paint achieved a higher temperature, the sponge utilized this heat better to improve the system’s efficiency. Despite being ranked third, the loofah still offers reasonable thermal efficiency compared to other materials, making it an effective option for improving the performance of solar desalination systems.

### Coating the absorber with sponge and CQDs

**Fig. 6:**
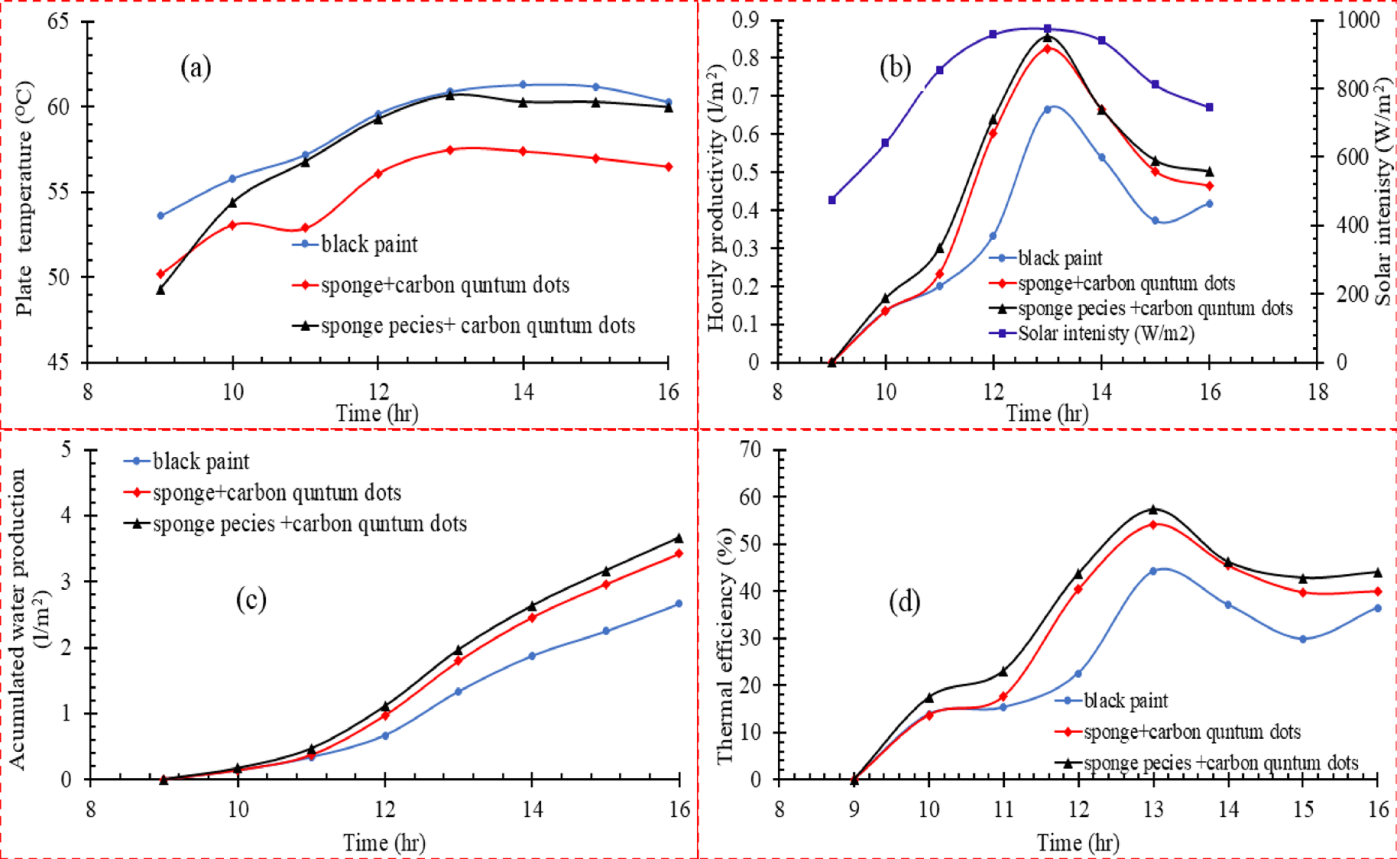
(**a**) Influence of absorber plate coating on **a**) the plate temperature, (**b**) water production, (**c**) Accumulated water production in the experimental period, and d) thermal efficiency in (scenario 3).

In the third scenario, the best results from the first and second scenarios are combined to analyze the properties of the sponge sprayed with CQDs compared to the black coating. Figure 6a shows that the absorption surface temperature of the black coating showed the highest temperature, reaching 61.3°C at the peak at 13:00. In contrast, sponge species and the sponge sprayed with CQDs showed slightly lower temperatures, reaching about 60.7°C and 57.5°C, respectively. Figure 6b**,** for the instantaneous productivity, shows that the sponge species sprayed with CQDs reached the highest instantaneous productivity of 0.85 L/m^2^/h, followed by the sponge with CQDs 0.82 L/m^2^/h. The black coating showed a maximum productivity of 0.66 L/m^2^/h. The sponge acts as an absorbent medium, allowing better water absorption and improved heat transfer between the surface and water. This results in an enhanced evaporation rate compared to black paint. The addition of quantum dots enhanced the efficiency due to improved radiation absorption and increased thermal transfer. From Fig. 6c for the cumulative productivity, the loofah sprayed with CQDs showed a cumulative productivity of 3.66 L/m^2^, followed by the sponge sprayed with a cumulative productivity of 3.42 L/m^2^. The black paint recorded the lowest cumulative productivity of 2.66 L/m^2^. The peak thermal efficiency of the loofah surface with CQDs was recorded as 57.28%, the sponge with CQDs 54.11%, and finally, the black paint 44.13%, as shown in Fig. 6d. The third scenario clearly showed that incorporating nanomaterials such as CQDs and GNPs with the sponge significantly improves the system performance compared to conventional black paint. Higher thermal performance and productivity are attributed to enhanced heat absorption efficiency and solar energy conversion into efficient heat for evaporation.

### Comparison with related works

Table 3 benchmarking the present work against recent enhanced tubular solar still studies, focusing on daily freshwater productivity and thermal efficiency. The selected studies are all experimentally validated, widely cited, and closely aligned with the enhancement strategies used in your manuscript (nanomaterials, porous/wicking media, hybrid approaches).

**Table 3:** Comparison of the present study with recent literature on enhanced tubular solar stills.

Ref	Enhancement strategy	Key materials used	Daily water productivity (L·m⁻^2^·day⁻^1^)	Thermal efficiency (%)	Main findings
Sharshir et al. ^26^	Nanocoated internal mushroom structure	Carbon black (25–75 g·m⁻^2^)	3.20	59.05	Nanocarbon coating significantly enhanced absorption and evaporation rate
Abdelaziz et al. ^27^	Wick + nanofluid + corrugated basin	CB nanoparticles + PCM	4.10	55–60	Synergistic effect of wick and nanomaterials improved productivity by ~ 89%
Sharshir et al. ^28^	Thin-film evaporation layer	Carbonized wood + CB	3.45	52.8	Optimized porous carbon layer enhanced thin-film evaporation
Sambare et al. ^29^	Sensible heat storage	Wire mesh / steel pieces	2.85	44.6	Wire mesh improved heat retention and evaporation
Essa et al. ^30^	Wick + nanocomposite	Jute / cotton + graphene–TiO_2_	4.20	58.7	Wick–nanocomposite coupling significantly boosted yield
Amin et al. ^33^	Heat exchanger + PCM + concentrator	Beeswax PCM + PTC	8.06	54.04	High productivity achieved using concentrator-assisted heating
Noman et al. ^36^	Bio-waste porous absorber	Custard apple seeds	3.10	46.2	Low-cost bio-porous media improved evaporation
Present study	Hybrid nanomaterial–porous coating	CQDs + sponge	3.66	57.28	Synergistic photothermal absorption and capillary-driven evaporation

## Conclusions

Three scenarios were applied to coat the absorber surface to test solar energy absorption and accelerate the evaporation process to improve the performance of the tubular solar still. The first scenario involves coating the absorber plate with carbon quantum dots (CQDs) and graphite nanoparticles (GNPs). The second scenario incorporates natural porous materials like a sponge and loofah, on the absorber plate. The third scenario combines CQDs and sponges and compares them with conventional black paint. In the first scenario, the results showed that nanocoatings outperformed black paint. The following key conclusions are drawn:

- Coating the absorber plate with carbon-based nanomaterials significantly enhanced photothermal performance. Compared to conventional black paint, the CQD-coated absorber increased cumulative freshwater productivity from 2.46 L/m^2^ to 3.21 L/m^2^, corresponding to an improvement of 30.49%, while peak thermal efficiency increased from 48.18% to 61.9%.
- Among the bio-based porous materials, the natural sponge outperformed loofah and black paint due to its superior capillary-driven evaporation. The sponge achieved a cumulative yield of 3.02 L/m^2^ and a thermal efficiency of 45.57%, compared to 2.67 L/m^2^ and 41.97% for black paint, respectively.
- The hybrid configuration combining carbon quantum dots with sponge exhibited the highest overall performance. This configuration achieved a maximum cumulative productivity of 3.66 L/m^2^, which is approximately 37.6% higher than that of the conventional black-painted absorber (2.66 L/m^2^). The corresponding peak thermal efficiency reached 57.28%, confirming the synergistic effect of enhanced optical absorption and porous-mediated thin-film evaporation.
- Instantaneous water productivity peaked at 0.85 L/m^2^·h for the hybrid CQDs–sponge surface, compared to 0.66 L/m^2^·h for the black-painted absorber, demonstrating a clear improvement in evaporation kinetics.

Potential future directions include exploring hybrid nanomaterial blends and integrating PCM layers beneath porous structures to enhance nighttime productivity. Also, aspects such as studying long-term durability and salt deposition on porous–nanomaterial surfaces, and investigating optical concentrators coupled with porous–nanomaterial absorbers.

## Data Availability

All required data are incorporated in the paper.
